# Descriptive Texts in Dog Profiles Associated with Length of Stay Via an Online Rescue Network

**DOI:** 10.3390/ani9070464

**Published:** 2019-07-20

**Authors:** Mizuho Nakamura, Navneet K. Dhand, Melissa J. Starling, Paul D. McGreevy

**Affiliations:** Sydney School of Veterinary Science, Faculty of Science, The University of Sydney, Sydney, NSW 2006, Australia

**Keywords:** dog, welfare, adoption, descriptors, temperament, breed

## Abstract

**Simple Summary:**

By investigating the descriptive text that constitutes an online profile of shelter dogs on PetRescue, the current study has identified personality adjectives that may influence the appeal of the four most common breeds available to pet adopters in Australia. If texts are likely to influence potential adopters’ decision-making, then the current findings reveal stark differences in desirable characteristics in canine companions, dependent on breed. The analysis showed that the presence of some terms and the absence of others had breed-specific associations with length of stay (LOS). For present terms, the shortest LOS among Australian cattle dogs were for those described as “active” (42.15 days); among Jack Russell terriers and Staffordshire bull terriers for those described as “gentle” (22.87 days and 32.23 days, respectively); and among Labrador retrievers for those described as quiet (22.18 days). For absent terms, the shortest LOS among Australian cattle dogs and Jack Russell terriers and Staffordshire bull terriers were for those *not* described as “energetic” (30.16 days, 19.58 days and 25.87 days, respectively); and among Labrador retrievers for those not described as “active” (18.79 days). This implies that breed may influence these expectations and what adopters are looking for in personality descriptors. Welfare shelters may wish to use these findings to modify their strategies when rehoming animals via online profiles.

**Abstract:**

To increase the public’s awareness of animals needing homes, PetRescue, Australia’s largest online directory of animals in need of adoption, lists animals available from rescue and welfare shelters nationwide. The current study examined the descriptions accompanying online PetRescue profiles. The demographic data and personality descriptors of 70,733 dogs were analysed for associations with LOS in shelters—with long stays being a potential proxy for low appeal. Univariable and multivariable general linear models of log-transformed LOS with personality adjectives and demographic variables were fitted and the predicted means back-transformed for presentation. Further analyses were conducted of a subset of the dataset for the four most common breeds (*n* = 20,198 dogs) to investigate if the influence of personality adjectives on the LOS differed by breed. The average LOS of dogs was 35.4 days (median 18 days) and was influenced by several adjectives. Across all breeds, the LOS was significantly shorter if the adjectives ‘make you proud’, ‘independent’, ‘lively’, ‘eager’ and ‘clever’ were included in the description. However, the LOS was longer if the terms ‘only dog’, ‘dominant’, ‘sensitive’ and ‘happy-go-lucky’ were included in the description. Some of the association of descriptors with relatively long LOS are difficult to explain. For example, it is unclear why the terms “obedient” and trainable” appear unappealing. The confidence adopters have in these terms and their ability to make the most of such dogs merits further exploration. As expected, the LOS differed in different breeds with the Labrador retrievers having the fastest adoption rate among the most common four breeds with an average LOS of 14.5 days. Breed had interactions with four personality adjectives (gentle, active, quiet and energetic) indicating that the adoption rate of dogs with these descriptors in their online PetRescue profiles differed by breed. This highlights an important knowledge gap, suggesting that potential adopters have differing expectations according to the breed being considered. Increased awareness of the breed-specific influence of personality adjectives on appeal to potential adopters, may enhance adoption success by allowing dogs with risk factors for low appeal to be promoted more intensively than high-appeal dogs.

## 1. Introduction

Dogs have been a part of human communities for thousands of years, assisting humans in hunting, guarding property and management of livestock. These traditional roles continue to this day, chiefly in rural areas, but dogs now also occupy more varied niches in contemporary society, primarily as companions. 

Thousands of companion dogs are abandoned or relinquished to welfare shelters every year (e.g., in Australia, the numbers are thought to exceed 200,000 [1]). Owner-related factors, such as moving to a new home with spatial restrictions or the birth of a child, alongside a lack of understanding and application of humane, effective dog training, result in high numbers of surrenders to welfare shelters [2,3]. Welfare and rescue shelters with available animals often post profiles online with photos and text, in the hope that the information provided will appeal to potential adopters [4,5,6]. Awareness of these animals may be further increased by sorting-house websites, which collate information on animals nationally. These initiatives allow members of the public to conduct general or specific searches (e.g., based on breed, age, sex and location) to find a potential companion. Furthermore, social media plays an important role in educating the general public about the number of dogs needing adoption and raising public awareness of individual animals available for adoption. Social media help to raise awareness of animals available for adoption, particularly those animals held by individual rescue or welfare groups. Compared with websites that can be time-consuming to maintain, social media accounts are relatively easy to create. Larger welfare organisations use social media to post photos and videos in “real time”, in addition to websites, and thus boost awareness of animals looking for a home.

PetRescue, Australia’s largest online sorting-house of homeless animals, invites shelters and rescue groups throughout Australia to list available animals on its website. Listings include breed, age, sex, photographs, and text describing the animal, prepared by foster carers, volunteers for rescue groups, or staff at shelters. Thus, PetRescue has an enormous database of historic pet profiles and listing statistics, presenting the opportunity to examine the effect that demographic factors and the words used to describe dogs in their online profiles may have had on their chances of adoption [7,8]. Animals with an extended length of stay (LOS), the number of days the animal is available for adoption before it is adopted, may be considered to be of low interest or appeal to members of the public looking to adopt a dog.

The purpose of the current study was to investigate the descriptive text within each animal’s profile and to identify risk factors involved in protraction of LOS. Demographic attributes (such as breed, age and sex) were also examined to investigate whether these factors reflect the putative appeal of animals on PetRescue as reflected by LOS. Descriptive text varies greatly in length, level of detail and whether it is written in the first or third person. It is less likely to be written with any emphasis on warnings about the animal because organisations aim to facilitate adoption. A study by Ley et al. [9] was used as the source of an array of personality dimensions that had previously been shown to resonate with dog owners. They kept some of the adjectives in their final list of personality dimensions. These dimensions facilitated the identification of personality descriptors in the current study.

By focussing on the use of personality adjectives relating to personality in these descriptions, we aimed to identify any breed-specific preferences of potential adopters when considering the animal’s advertised characteristics. A potential benefit of the resultant information may lie in its use to optimise the exposure of animals of low appeal as assessed by LOS.

## 2. Materials and Methods

### 2.1. Selection

The sample population for this study was all dogs listed on the PetRescue website from 2004 (when PetRescue was founded) until late March 2013 (when this study commenced). There were 122,634 dogs listed on the website over this nine-year period. As this study investigated variables relative to adoption speed and focussed solely on rehomed dogs, we excluded “removed” and “active” dogs from the dataset, leaving a pool of 101,397. “Removed” dogs were those that had had an individual listing on the PetRescue website but had been removed by the independent rescue groups for unspecified reasons. “Active” dogs were newly listed on the website and therefore still available for adoption. The eventual LOS of these “active” dogs was therefore unknown, so these dogs were eliminated from the study. Furthermore, numerous dogs had data missing on LOS. After these dogs had been excluded, the total number available for this study was 70,733 dogs. Demographic data on the 70,733 dogs were provided in the data source. Extensive filtering and scanning were performed in a Microsoft Excel spreadsheet to locate and identify the keywords/descriptors throughout the data set. This dataset was used for primary analyses reported in this manuscript.

From the final pool of 70,733 dogs, a data subset comprised of the four most common breeds only: the Staffordshire bull terrier, Australian cattle dog, Jack Russell terrier and Labrador retriever (total *n* = 20,198) were selected for additional analysis to explore any relationships between breed, descriptive text and LOS. Many dogs of these breeds were listed onto the website with various spellings and truncations (such as “Staffy” for Staffordshire bull terrier, “Lab” for Labrador retriever, “Jack Russell” for Jack Russell terrier and “Cattle dog” for Australian cattle dog). Dogs listed with these derivative labels were also included in the final breed dataset. 

PetRescue owns and provided the data in a Microsoft Excel^TM^ spreadsheet (Microsoft Corporation, One Microsoft Way, Redmond, WA, USA). Statistical analyses were conducted using the SAS statistical program (SAS 9.4^©^ 2002–2012 by SAS Institute Inc., Cary, NC, USA).

### 2.2. Outcome Variable 

As the putative risk factors of low appeal animals were of interest, the date listed (on the website) and date removed (adopted via the individual welfare groups) were used to estimate LOS. It is acknowledged that these dates may have been inaccurate, particularly if listings were not updated promptly to record the dog’s adoption. The retrospective dataset we used meant that it was not possible to determine the accuracy of date reporting. Therefore, the use of dates in this way is considered a proxy of LOS rather than an absolute measure of LOS. A minimum of zero meant that the LOS was less than one day. LOS was log-transformed to meet the assumptions of normality and homoscedasticity. All the analyses reported in the manuscript are based on the log-transformed variable, unless indicated otherwise.

### 2.3. Explanatory Variables 

Demographic information on each dog (breed, age and sex) as well as accompanying descriptive text was explored for any associations with LOS in the full dataset of 70,733 dogs. Age was used as a continuous explanatory variable in the analyses whereas all other explanatory variables were categorical. 

A total of 45 terms comprised 39 personality adjectives from Ley et al. [9], and a further six that were recurring themes that emerged after extensive filtering of the data set. Frequency and summary statistics of these recurring terms were conducted. Personality adjectives were sorted into dimensions comprising suites of traits to further filter the raw data. The adjectives (*n* = 45) eventually categorised were assigned to the clusters of personality adjectives that had previously been shown to resonate with dog owners on the basis of five dimensions of personality (energetic/extroverted, self-assured/motivated, nervous/sensitive, responsiveness to training, friendliness/sociability) [9]. Ley et al. identified these dimensions through a process of refining the list of adjectives used to describe dogs and first eliminating words that were ambiguous or did not apply to dogs, or applied to nearly all dogs, and then using a Principal Component Analysis to reveal clusters of adjectives that described the same broader dimension. The resulting lists of dimensions and adjectives were later validated [9]. For the current analysis, personality adjectives (*n* = 45) were used. Also recurring themes in the online descriptions, such as “no cats”, “no children”, “only dog”, “not dominant”, “not hyperactive” and “make you proud” were evident within the data and were also used as explanatory variables along with the personality adjectives.

### 2.4. Statistical Analyses 

Summary statistics and graphical summaries for LOS and frequency tables for categorical variables were created to map the distribution of variables for the complete dataset. Summary statistics and box-and-whisker plots for LOS by demographic variables and personality adjectives were then created to make a preliminary evaluation of associations between them. 

All demographic variables (age, sex and breed) and personality adjectives were then tested for their unconditional association with log-transformed LOS using univariable general linear regression approach using the entire dataset of 70,733 dogs. Explanatory variables with *p*-value < 0.2 were then tested in multivariable models built using a forward stepwise approach to explore associations of log-LOS with demographic and personality variables, after adjusting for each other. Breed and age had to be excluded from these analyses due to their large number of missing values. The assumptions of normality and homoscedasticity were tested using residual diagnostics and were met for log-transformed LOS. Log-transformed LOS means predicted by the final model were back-transformed. Back-transformation converts log-transformed LOS means into geometric means on the original scale, i.e., from log days to days which are easier to interpret. Similarly, the differences between means on the log scale were back-transformed. The difference of means on the log scale conveniently become a ratio of the means on the original scale. 

The analysis of the sub-population of the four most common breeds examined breed as an explanatory variable for measuring differences in log-transformed LOS. This secondary analysis on the partial dataset was completed to focus on how breed may interact with other variables to predict log-transformed LOS. Breed was added the final model built using the entire dataset and non-significant variables were deleted. Interactions of significant variables were tested with breed. Similar to above, predicted means and their differences were calculated and back-transformed for presentation. 

## 3. Results

### 3.1. Descriptive Results

Summary statistics of age and LOS, the only quantitative variables in the dataset, are presented in Table 1. 

The median age of dogs in the dataset was 18.0 months and median LOS was 18.05 days. Female dogs in this study had a slightly shorter LOS than male dogs. Summary statistics of the outcome (LOS) by the categorical explanatory variables (personality adjectives) are presented in Table 2 to illustrate the contrast in LOS when these adjectives were present or absent from the text of profiles. 

The resultant adjective table (Table 2) was divided into five main dimensions (energetic/extroverted, self-assured/motivated, responsiveness to training, friendly/sociable and nervous sensitivity). These five dimensions had different numbers of adjectives per dimension: energetic/extroverted (10 adjectives), self-assured/motivated (seven adjectives), responsiveness to training (10 adjectives), friendly/sociable (eight adjectives) and nervous sensitivity (six adjectives). Friendly/sociable had the greatest frequency in the dataset at 37 percent (Table 2). Responsiveness to training had the lowest frequency (three percent) in the dataset, followed by nervous sensitivity (six percent), self-assured/motivated (10 percent) and energetic/extroverted (32 percent). The traits and combination of “active”, “energetic”, “quiet” and “gentle” were the most commonly observed in the current study. These personality adjectives also fall into the two highest frequency categories as outlined above.

### 3.2. General Text Analysis

Analyses relative to log-transformed LOS were conducted for 70,733 dogs. Associations of age and sex with log-transformed LOS were investigated in univariable analyses but age and breed were excluded from multivariable analyses due to a large number of missing values. Sex was included in multivariable analyses and was significant in the final model. The assumptions of normality and homoscedasticity were met for log-transformed LOS. The results of the final model presented in Table 3 include predicted mean LOS if an adjective was present and absent and the ratio of the two means. For example, average LOS was 37.7 days if the adjective “dominant” was present, compared to 25.9 days if it was absent. Thus, the average LOS of dogs with this adjective present in their online profile was 1.46 times that of dogs without this adjective (presented as a ratio). On the other hand, the average LOS if the descriptor “make you proud” was present was about one third (0.36) than that of the dogs where this adjective was not present. 

It is acknowledged that dogs may contribute data to more than one group. Absence of the “not dominant” and “not hyperactive” personality adjectives does not suggest dominance and/or hyperactivity in the animal. A minimum of zero means that the LOS was less than one day.

### 3.3. Top Four Breeds Text Analysis

In the analysis of breeds and LOS, Labrador retrievers had the shortest median LOS of 14.5 days and the fastest speed of adoption among the common breeds in this study. They were followed by Jack Russell terriers, with a median LOS of 16.2 days, Staffordshire bull terrier (16.5 days) and Australian cattle dogs (21.4 days). Further analyses were conducted for top four breeds by adding breed to the model presented in Table 3 and then excluding non-significant variables and testing interactions of breed with all significant variables. Only the results of the significant interactions are presented in Table 4 as the association of other variables were not very different than the results presented in Table 3. Significant interactions were observed for gentle, active, quiet and energetic, meaning that the LOS for these adjectives varied by breed. For example, the LOS was longer if the adjective gentle was included in the profile for Australian cattle dogs (44.75 vs. 36.71 days) but the reverse was true for Jack Russell terriers (22.87 vs. 24.9 days). 

Breed was added to the final model presented in Table 3 for the entire data and non-significant variables were excluded (see Table 5). The interactions of breed with all the significant personality adjectives were tested and retained if significant (presented in Table 6). The results presented are back-transformed means and their ratios and are adjusted for sex. 

## 4. Discussion

The current study explored a total of 45 personality adjectives, 39 of which align with Ley et al. [9] across five categories. Many of these categories were associated with average LOS (Table 2). The category of friendly/sociable was the most commonly represented, with an appearance in 37 percent of profiles, followed by the category of energetic/extroverted (32 percent). This is unsurprising because, in most cases, the text or description of a dog for an online profile is largely intended to pique adopters’ interest in the dogs.

Dogs with an LOS of zero would suggest that the dog was listed on and removed from the online directory on the same day, i.e., adopted straight away. It would be unwise to assume that the adopter saw the online profile and proceeded to visit the shelter and acquire the dog on that day. With this is mind, we acknowledge that, for all dogs especially those with an LOS of zero, the online advertisement may not have been the sole effect on potential adopters. We accept that LOS is merely a proxy for the total time a given dog spends at the shelter. One could argue that a better term for this variable may be “length of online advertisement”.

Labrador retrievers had the shortest median LOS followed by Jack Russell terriers, Staffordshire bull terrier and Australian cattle dogs. This contrasts with the findings of Protopopova et al. [10] who reported that sporting breeds (such as the Labrador retriever and golden retriever) had the longest LOS in shelters, while so-called ratters (such as Jack Russell terriers, fox terriers and dachshunds) had the shortest LOS. Brown et al. [11] and Svoboda and Hoffman [12] found that puppies (0–6 months) had the shortest LOS, and that the LOS increased as the dogs got older. Similar results were found in the current study.

Four personality adjectives in the sample population of Staffordshire bull terriers, Australian cattle dogs, Jack Russell terriers and Labrador retrievers (“active”, “gentle”, “energetic” and “quiet”), had a significant association with LOS. Of the personality adjectives significantly associated with LOS, the five descriptive terms associated with the shortest LOS were “restless” (14.32 days), “make you proud” (16.19 days), “independent” (23.44 days) “lively” (25.71 days) and “eager” (26.52 days). The use, in an online profile, of the term “make you proud” may suggest a hard-working, eager and trainable dog, which may be appealing and desirable to prospective owners. The same might be said for “eager” but “restless” and “independent” present a markedly different picture. “Restlessness” was associated with the shortest LOS in dogs, implying that it is a highly desirable trait. That said, it must be noted that the frequency of “restlessness” in the descriptive texts was very low (37 counts) and suggests that this result should be interpreted with caution. It may be that a “restless” dog suggests one that is uncomfortable in its surroundings, and in need of rescuing—thus appealing to the possible primary motivation of many people visiting PetRescue to help a dog in need. An alternative explanation is that “restless” dogs crave physical activity. In a study by Starling et al. [13], active dogs (recorded alongside playful and energetic dogs) were listed as the most sought-after (42.2 percent). It is unclear what the appeal of “independent” may be. Perhaps it serves as a proxy for “not distressed when left alone”, and so may appeal to those who work full-time and will not be home with the dog during most working days.

Some of the association of descriptors with relatively long LOS are difficult to explain. For example, it is unclear why the terms “obedient” and trainable” appear unappealing. The confidence adopters have in these terms and their ability to make the most of such dogs merits further exploration.

It must be noted that the number of appearances of these personality adjectives in descriptive texts varied greatly (e.g., “restless” had 37 counts and “make you proud” had 306 counts). For this reason, we again cannot report an absolute association with personality adjectives and LOS. After performing the analysis of personality adjectives per breed, several statistically significant associations emerged. The analysis showed that the presence of some terms and the absence of others had breed-specific associations with LOS. For present terms, the shortest LOS among Australian cattle dogs were for those described as “active” (42.15 days); among Jack Russell terriers and Staffordshire bull terriers for those described as “gentle” (22.87 days and 32.23 days, respectively); and among Labrador retrievers for those described as quiet (22.18 days). For absent terms, the shortest LOS among Australian cattle dogs and Jack Russell terriers and Staffordshire bull terriers were for those not described as “energetic” (30.16 days, 19.58 days and 25.87 days, respectively); and among Labrador retrievers for those not described as “active” (18.79 days). These findings raise questions about the expectations adopters have of different breeds, and why they might favour them. For example, it is possible that people who wish to own an Australian cattle dog favour this working breed because its activity and stamina accord with plans to boost or maintain their physical activity. In contrast, Jack Russell terriers may be preferred by those seeking dogs of a conveniently small size, while Labrador retrievers and Staffordshire bull terriers may be more favoured by those seeking dogs with bold temperaments [13].

Some dogs in the current study were described and portrayed as not being a particular way or not having a given personality trait. Examples include “not dominant” and “not hyperactive”. This may indicate that these personality traits are considered undesirable among potential adopters. Dogs profiled with “dominant” or “hyperactive” in their descriptive texts had a significant increase in LOS. 

Protopopova and Wynne found that 81.8 percent of 248 respondents cited desirable behaviour as a major reason for adoption and that 31.3 percent of adopters preferred friendly dogs [14]. In the same study, 12.5 percent of respondents valued dogs that were cat-, dog- and child-friendly. Descriptions within the “friendly/sociable” category were the most frequently encountered in the current study. This presumably reflects the perceived importance of friendliness by potential adopters, a perception that is supported by previous studies. Friendliness is known to be one of the key behavioural attributes deemed “ideal” by dog owners [15]. Friendliness toward people, other dogs and other animals has been shown to influence the likelihood of adoption [16]. 

The study had a large sample size and hence had enormous power to detect even minor differences in LOS means between groups. As a result, some of the variables with very small differences in means for the presence vs. the absence of a descriptor became statistically significant (e.g., 30.09 vs. 32.45 days for “clever”; Table 3). We acknowledge that the variables with small differences, even though statistically significant, may not be biologically meaningful. Certainly, they would not be as biologically important as variables with larger differences (e.g., 18.84 vs. 51.84 for “make you proud”; Table 3). Therefore, in addition to evaluating *p*-values, the actual means or their ratios should be considered while interpreting associations, and the statistical significance of variables with small differences should be interpreted with caution.

The results of the current study suggest that the way in which a dog is described (use of personality adjectives) affects LOS and ultimately adoption. Depending on the prospective owner’s domestic circumstances and lifestyle, the attributes valued in a canine companion vary greatly. Dog profiles with keywords that include “good with children” will appeal to families with children, increasing interest in the dog and potentially leading to a visit to the shelter to establish whether the dog interacts well with the prospective adopter’s children. It is likely that the initial interest in and awareness of a potentially suitable dog arises online, rather than in-house. These days, many prospective owners conduct some research online or contact the welfare organisation directly for more information about available dogs; before travelling to the shelter. It seems appropriate to acknowledge that, when described authentically, some dogs are not attractive to prospective adopters. For such dogs, there is merit in diverting resources to modifying their behaviour and raising awareness of their availability for adoption among the kinds of people to whom they are attractive, if that were known.

## 5. Conclusions

By investigating the descriptive text within the online profiles of shelter dogs listed on PetRescue, the current study has identified personality adjectives that may influence the appeal of the four most common breeds available to prospective adopters in Australia. The personality adjectives that were significantly associated with LOS differed with breed, suggesting that prospective adopters’ preferences may be breed-specific. It may be that welfare shelters can use these findings to modify their strategies when rehoming animals using online profiles. 

## Figures and Tables

**Table 1:** Summary statistics for quantitative variables (by sex) in the complete dataset based on 70,733 observations.

Variable	Group	Arithmetic Mean	SD	Minimum	Q1	Median	Q3	Maximum	Num
LOS	Overall	35.4	54.8	0	6.9	18.0	41.9	1409.0	70,733
(days)	Male	36.9	51.6	0	6.9	18.8	43	1163.9	37,733
	Female	33.7	51.7	0	6.7	17.4	40.6	1409.0	33,000
Age	Overall	27.2	29.1	0.5	6.0	18.0	36.0	216	59,017
(months)	Male	27.4	28.8	0.5	6.0	18.0	36.0	216	31,477
	Female	27.0	29.4	0.5	5.0	18.0	36.0	192	27,540

LOS: Length of stay; SD: Standard deviation; Q1: First quartile; Q3: Third quartile; Number used: Number of observations excluding missing values; Number Total: Total number of observations including missing values.

**Table 2:** Mean LOS (in days) per adjective and percentage representation (%) of frequency in each of five personality dimensions (comprising variables, *n* = 39, sourced from Ley et al., 2009) and six separate themes recurring in the current database of 70,733 dogs of all breeds. Numbers marked with (*) represent insufficient data (<1%) due to low frequency within dimension. Percentages appear for adjectives and how commonly dimensions were represented by each of their associated adjectives.

Explanatory Variable	Category	Num	Mean LOS	SD	Min.	Q1	Median	Q3	Max.
Dimension 1: Energetic/Extroverted									
Active	Absent	61,780	34.7	54.24	0	7	18	41	1409
	Present	8953	40.39 (39%)	58.15	0	8	21	49	859
Quiet	Absent	63,986	35.09	54.57	0	7	18	41	1164
	Present	6747	38.61 (29%)	56.69	0	9	22	47	1409
Energetic	Absent	68,234	35.13	54.45	0	7	18	42	1409
	Present	2499	43.5 (11%)	62.62	0	8	22	51	846
Eager	Absent	68,504	35.41	54.74	0	7	18	42	1409
	Present	2229	35.72 (10%)	56.18	0	5	18	43	965
Lively	Absent	69,721	35.46	54.87	0	7	18	42	1409
	Present	1012	32.91 (4%)	48.06	0	6	17	42	587
Excitable	Absent	70,004	35.45	54.73	0	7	18	42	1409
	Present	729	33.41 (3%)	59.39	0	7	16	37	965
Enthusiastic	Absent	70,278	35.4	54.72	0	7	18	42	1409
	Present	455	39.08 (2%)	63.15	0	8	20	44	652
Exuberant	Absent	70,500	35.44	54.83	0	7	18	42	1409
	Present	233	31.08 (1%)	35.49	0	8	17	44	248
Hyperactive	Absent	70,652	35.41	54.79	0	7	18	42	1409
	Present	81	49.67 *	50.23	0	16	35	67	266
Restless	Absent	70,695	35.44	54.79	0	7	18	42	1409
	Present	38	9.95 *	16.43	0	1	2.5	11.5	71
Dimension 2: Self-assured/Motivated									
Intelligent	Absent	67,682	35.15	54.54	0	7	18	42	1409
	Present	3051	41.46 (45%)	59.7	0	9	22	51	965
Obedient	Absent	68,638	35.24	54.8	0	7	18	42	1409
	Present	2095	41.4 (31%)	53.91	0	10	23	50	459
Clever	Absent	69,646	35.43	54.63	0	7	18	42	1164
	Present	1087	35.04 (16%)	63.58	0	6	17	45	1409
Attentive	Absent	70,431	35.42	54.79	0	7	18	42	1409
	Present	302	35.79 (4%)	52.91	1	7.25	18	43	590
Trainable	Absent	70,580	35.41	54.79	0	7	18	42	1409
	Present	153	41.19 (2%)	50.84	0	12	26	51	366
Reliable	Absent	70,651	35.42	54.79	0	7	18	42	1409
	Present	82	41.87 (1%)	50.19	1	11.25	25.5	55.5	248
Biddable	Absent	70,688	35.43	54.8	0	7	18	42	1409
	Present	45	32.27 *	29.28	3	10	22	49	122
Dimension 3: Responsiveness to training									
Proud	Absent	70,203	35.48	54.81	0	7	18	42	1409
	Present	530	27.42 (27%)	50.68	0	4	12	31	590
Independent	Absent	70,211	35.48	54.88	0	7	18	42	1409
	Present	522	28.03 (26%)	39.1	0	4	13	39	321
Dominant	Absent	70,211	35.31	54.69	0	7	18	42	1409
	Present	522	50.85 (26%)	63.92	0	14	31	59	541
Thorough	Absent	70,509	35.39	54.79	0	7	18	42	1409
	Present	224	45.08 (11%)	50	0	11	28.5	59	315
Determined	Absent	70,645	35.42	54.77	0	7	18	42	1409
	Present	88	36.9 (4%)	61.48	1	6	17	42	456
Assertive	Absent	70,656	35.42	54.78	0	7	18	42	1409
	Present	77	35.1 (4%)	59.05	0	6	21	42	457
Nosey	Absent	70,726	35.43	54.78	0	7	18	42	1409
	Present	7	11.14 *	9.67	0	6	9	13.5	30
Tenacious	Absent	70,730	35.42	54.78	0	7	18	42	1409
	Present	3	34 *	22.61	15	21.5	28	43.5	59
Opportunistic	Absent	70,731	35.42	54.78	0	7	18	42	1409
	Present	2	49 *	25.46	31	40	49	58	67
Dimension 4: Friendly/Sociable									
Friendly	Absent	57,181	34.91	54.64	0	7	18	41	1164
	Present	13,552	37.6 (58%)	55.31	0	8	20	45	1409
Gentle	Absent	61,192	34.77	54.34	0	7	18	41	1409
	Present	9,541	39.63 (36%)	57.39	0	9	22	47	870
Sociable	Absent	69,570	35.46	54.91	0	7	18	42	1409
	Present	1,163	33.22 (4%)	46.26	0	8	18	40.5	564
Relaxed	Absent	69,756	35.43	54.84	0	7	18	42	1409
	Present	977	34.91 (4%)	50.72	0	6	19	42	514
Happy go lucky	Absent	70,556	35.4	54.74	0	7	18	42	1409
	Present	177	46.29 *	69.49	0	14	28	51	678
Easy going	Absent	70,558	35.41	54.81	0	7	18	42	1409
	Present	175	39.41 (4%)	42.55	0	11.5	23	47	238
Non aggressive	Absent	70,715	35.42	54.78	0	7	18	42	1409
	Present	18	52.78 *	70.43	1	16.25	29.5	37.75	231
Dimension 5: Nervous/Sensitive									
Timid	Absent	68,628	35.27	54.77	0	7	18	42	1409
	Present	2105	40.52 (52%)	55.04	0	9	23	49	666
Submissive	Absent	69,950	35.36	54.79	0	7	18	42	1409
	Present	783	41.16 (19%)	54	0	11	25	51	577
Nervous	Absent	70,158	35.4	54.75	0	7	18	42	1409
	Present	575	38.98 (14%)	58.15	0	8	21	45	652
Sensitive	Absent	70,376	35.32	54.66	0	7	18	42	1409
	Present	357	55.55 (9%)	72.17	0	10	32	71	590
Fearful	Absent	70,617	35.41	54.76	0	7	18	42	1409
	Present	116	46.72 (3%)	66.01	0	7	22.5	47.25	317
Cautious	Absent	70,640	35.41	54.72	0	7	18	42	1409
	Present	93	45.2 (2%)	89.15	0	4	14	48	652
Recurring themes (*n* = 6)									
Only dog	Absent	68,626	34.98	54.43	0	7	18	41	1409
	Present	2107	50.04	63.51	0	13	30	63	1025
Make you proud	Absent	70,426	35.51	54.83	0	7	18	42	1409
	Present	307	15.74	37.95	0	3	7	16	590
No children	Absent	70,429	35.38	54.64	0	7	18	42	1409
	Present	304	45.99	81.35	0	8	22	49.5	841
No cats	Absent	70,492	35.39	54.78	0	7	18	42	1409
	Present	241	45.29	54.92	0	11	25	57	339
Not hyperactive	Absent	70,699	35.41	54.78	0	7	18	42	1409
	Present	34	56.88	61.09	1	16.5	37	60.75	266
Not dominant	Absent	70,709	35.42	54.79	0	7	18	42	1409
	Present	24	44.04	38.91	14	18.75	36.5	51.25	205

SD: Standard deviation; Q1: First quartile; Q3: Third quartile; Num: Total number of observations.

**Table 3:** Results of the final multivariable model of the association of individual personality adjectives (whether present or absent) addressed in descriptive texts (*n* = 24) relative to LOS of 70,733 shelter dogs.

Personality Adjective	Predicted Back-Transformed LOS Means		Ratio of Means (95% CI)	*p*-Value
	Present	Absent		
Only dog	39.21	24.91	1.57 (1.49, 1.66)	<0.001
Gentle	33.39	29.25	1.14 (1.11, 1.17)	<0.001
Active	33.59	29.08	1.16 (1.12, 1.19)	<0.001
Quiet	33.46	29.19	1.15 (1.11, 1.18)	<0.001
Friendly	33.29	29.33	1.14 (1.11, 1.16)	<0.001
Make you proud	18.84	51.84	0.36 (0.29, 0.45)	<0.001
Obedient	34.82	28.05	1.24 (1.18, 1.31)	<0.001
Dominant	37.72	25.89	1.46 (1.31, 1.62)	<0.001
Energetic	34.45	28.35	1.22 (1.16, 1.28)	<0.001
Intelligent	33.24	29.38	1.13 (1.08, 1.18)	<0.001
Timid	34.2	28.55	1.2 (1.14, 1.26)	<0.001
Sensitive	37.2	26.26	1.42 (1.25, 1.61)	<0.001
Restless	16.16	60.44	0.27 (0.18, 0.39)	<0.001
Proud	35.22	27.73	1.27 (1.08, 1.49)	0.003
Submissive	33.6	29.07	1.16 (1.06, 1.26)	<0.001
Independent	26.61	36.7	0.73 (0.65, 0.81)	0.001
No cats	36.52	26.74	1.37 (1.17, 1.59)	<0.001
Thorough	35.92	27.19	1.32 (1.13, 1.55)	<0.001
Happy-go-lucky	37.26	26.21	1.42 (1.19, 1.7)	<0.001
Hyperactive	35.93	27.18	1.32 (1.01, 1.72)	0.039
Trainable	34.89	27.99	1.25 (1.03, 1.51)	0.025
Eager	30.05	32.5	0.92 (0.88, 0.97)	0.036
Lively	29.26	33.37	0.88 (0.81, 0.95)	<0.001
Clever	30.09	32.45	0.93 (0.86, 1)	0.044

Only significant (*p* < 0.05) associations are presented and the results are adjusted for sex.

**Table 4:** Summary statistics of LOS for the four most common breeds (*n* = 20,198 dogs).

Breed	Arithmetic Mean	SD	Minimum	Q1	Median	Q3	Maximum	Num
Australian cattle dog	41.1	57.1	0	7.7	21.4	51.8	570.5	3275
Jack Russell terrier	34.4	53.3	0	6.8	16.2	38.4	662.1	4200
Labrador retriever	27.6	40.6	0	5.1	14.5	33.3	524.3	4105
Staffordshire bull terrier	33.4	56.2	0	6.6	16.2	37.8	1409.0	8618

**Table 5:** The results of the second final multivariable model of LOS restricted to data for only the four most common breeds (*n* = 20,198 dogs).

Personality Adjective	Predicted Back-Transformed LOS Means		Ratio of Means (95% CI)	*p*-Value
	Present	Absent		
Only dog	35.17	22.38	1.57 (1.41, 1.75)	<0.001
Friendly	30.68	25.65	1.20 (1.15, 1.25)	<0.001
Make you proud	16.19	48.52	0.33 (0.23, 0.47)	<0.001
Obedient	32.45	24.25	1.34 (1.22, 1.47)	<0.001
Dominant	35.96	21.88	1.64 (1.36, 1.99)	<0.001
Intelligent	31.02	25.37	1.22 (1.12, 1.33)	<0.001
Timid	30.96	24.06	1.22 (1.10, 1.35)	0.0002
Sensitive	32.70	26.26	1.36 (1.06, 1.74)	0.0158
Restless	14.32	54.97	0.26 (0.15, 0.45)	<0.001
Proud	33.39	23.57	1.42 (1.07, 1.87)	0.0147
Independent	23.44	33.58	0.70 (0.57, 0.85)	0.0004
No cats	34.16	23.04	1.48 (1.11, 1.98)	0.0078
Thorough	34.13	23.06	1.48 (1.03, 2.13)	0.0346
Happy-go-lucky	33.84	23.26	1.45 (1.08, 1.95)	0.0127
Eager	26.52	29.68	0.89 (0.82, 0.98)	0.0126
Lively	25.71	30.61	0.84 (0.73, 0.97)	0.0162

**Table 6:** Interactions between breed and personality adjectives based on the final model of LOS for dogs (*n* = 20,198 dogs) of the four most common breeds.

Personality Adjective		Predicted Back-Transformed LOS Means				*p*-Value
		Breed				
		Australian Cattle Dog	Jack Russell Terrier	Labrador Retriever	Staffordshire Bull Terrier	
Gentle	Present	44.75	22.87	23.42	32.23	<0.01
	Absent	36.71	24.90	20.14	26.96	<0.01
	Ratio (95% Cl)	1.22 (1.07, 1.39)	0.92 (0.8, 1.05)	1.16 (1.05, 1.29)	1.2 (1.1, 1.3)	
Active	Present	42.15	23.84	25.11	32.43	<0.01
	Absent	38.97	23.90	18.79	26.8	<0.01
	Ratio (95% Cl)	1.08 (0.98, 1.2)	1.34 (0.89, 1.11)	1.34 (1.2, 1.49)	1.21 (1.12, 1.31)	
Quiet	Present	45.44	23.21	22.18	32.64	<0.01
	Absent	36.15	24.54	21.27	26.63	<0.01
	Ratio (95% Cl)	1.26 (1.08, 1.46)	0.95 (0.83, 1.08)	1.04 (0.9, 1.21)	1.23 (1.11, 1.35)	
Energetic	Present	54.46	29.09	22.68	33.60	<0.01
	Absent	30.16	19.58	20.8	25.87	<0.01
	Ratio (95% Cl)	1.81 (1.45, 2.25)	1.49 (1.23, 1.79)	1.09 (0.92, 1.29)	1.3 (1.14, 1.48)	

## References

[1] Chua D., Rand J., Morton J. (2017). Surrendered and stray dogs in Australia—Estimation of numbers entering municipal pounds, shelters and rescue groups and their outcomes. Animals.

[2] Poulsen A.H., Lisle A.T., Phillips C.J.C. (2010). An evaluation of a behaviour assessment to determine the suitability of shelter dogs for rehoming. Vet. Med. Int..

[3] Mornement K.M., Coleman G.J., Toukhsati S., Bennett P.C. (2010). A review of behavioural assessment protocols used by Australian animal shelters to determine the adoption suitability of dogs. J. Appl. Anim. Welf. Sci..

[4] Fratkin J.L., Baker S.C. (2013). The role of coat colour and ear shape on the perception of personality in dogs. Anthrozoos.

[5] Marder A., Duxbury M.M. (2008). Obtaining a pet: Realistic expectations. Small Anim. Pract..

[6] Lampe R., Witte T. (2015). Speed of dog adoption: Impact of online photo traits. J. Appl. Anim. Welf. Sci..

[7] Wells D., Hepper P.G. (1992). The behaviour of dogs in a rescue shelter. Anim. Welf..

[8] Luescher A.U., Medlock R.T. (2009). The effects of training and environmental alterations on adoption success of shelter dogs. Appl. Anim. Behav. Sci..

[9] Ley J., Bennett P., Coleman G. (2008). Personality dimensions that emerge in companion canines. Appl. Anim. Behav. Sci..

[10] Protopopova A., Gilmour A.J., Weiss R.H., Shen J.Y., Wynne C.D.L. (2012). The effects of social training and other factors on adoption success of shelter dogs. Appl. Anim. Behav. Sci..

[11] Brown W.P., Davidson J.P., Zuefle M.E. (2013). Effects of phenotypic characteristics on the length of stay of dogs at two no kill animal shelters. J. Appl. Anim. Welf. Sci..

[12] Svoboda H.J., Hoffman C.L. (2015). Investigating the role of coat colour, age, sex, and breed on outcomes for dogs at two animal shelters in the United States. Anim. Welf..

[13] Starling M.J., Branson N., Thomson P.C., McGreevy P.D. (2013). Age, sex and reproductive status affect boldness in dogs. Vet. J..

[14] Protopopova A., Wynne C.D.L. (2014). Adopter-dog interactions at the shelter: Behavioural and contextual predictors of adoption. Appl. Anim. Behav. Sci..

[15] King T., Marston L.C., Bennett P.C. (2009). Describing the ideal Australian companion dog. Appl. Anim. Behav. Sci..

[16] Siettou C., Fraser I.M., Fraser R.W. (2014). Investigating some of the factors that influence “consumer” choice when adopting a shelter dog in the United Kingdom. J. Appl. Anim. Welf. Sci..

